# Cell Models to Study Regulation of Cell Transformation in Pathologies of Retinal Pigment Epithelium

**DOI:** 10.1155/2014/801787

**Published:** 2014-08-07

**Authors:** Alla V. Kuznetsova, Alexander M. Kurinov, Maria A. Aleksandrova

**Affiliations:** N.K. Koltsov Institute of Developmental Biology, Russian Academy of Sciences, ul. Vavilova 26, Moscow 119334, Russia

## Abstract

The retinal pigment epithelium (RPE) plays a key role in the development of many eye diseases leading to visual impairment and even blindness. Cell culture models of pathological changes in the RPE make it possible to study factors responsible for these changes and signaling pathways coordinating cellular and molecular mechanisms of cell interactions under pathological conditions. Moreover, they give an opportunity to reveal target cells and develop effective specific treatment for degenerative and dystrophic diseases of the retina. In this review, data are presented on RPE cell sources for culture models, approaches to RPE cell culturing, phenotypic changes of RPE cells *in vitro*, the role of signal pathways, and possibilities for their regulation in pathological processes.

## 1. Introduction

The retinal pigment epithelium (RPE) has a number of important physiological functions, including the maintenance of the structure and functions of photoreceptors and the blood-retinal barrier. Although RPE forms a dense monolayer of nonproliferating cells, it is capable of transformation into other cell types, with this capability varying in the series of vertebrates. In adult newts, for example, central RPE cells and low-differentiated cells of the peripheral growth zone account for regeneration of the retina [1–4]. After the surgical removal of the retina, RPE cells dedifferentiate, lose pigment, and proliferate; thereafter, part of cells recover RPE differentiation, while another part transdifferentiate into neural retinal cells [5]. Unlike in lower vertebrates, in which the ability to regenerate the retina via transdifferentiation is lifelong, its regeneration in some mammals is possible only during the embryonic period [6], whereas RPE plasticity retained in adults is responsible for a variety of ocular pathologies. Thus, RPE damage in humans initiates processes similar to its transdifferentiation in urodeles: RPE cells lose pigment, proliferate, migrate, and differentiate into different cell types, expressing appropriate markers (atypical of RPE) [7], but fail to produce a new functional retina. In pathological cases, RPE cells often transdifferentiate not into neural retinal cells but into fibroblast-like cells, which, in the “wet” (exudative) form of age-related macular degeneration (AMD), are involved in the formation of subretinal (choroidal) neovascular membrane [8–10]. In pathologies such as proliferative vitreoretinopathy (PVR) and proliferative diabetic retinopathy, transformed RPE cells contribute to the formation of epiretinal membranes [10–12], with consequent visual impairment.

There is no universally accepted term for what occurs with RPE cells* in vivo* under pathological conditions. Thus, phenotypic changes observed in RPE cells are referred to as “metaplasia” [13], “transformation” [9, 10, 14], “epithelial-mesenchymal transdifferentiation” [9], or “epithelial-mesenchymal transition” (EMT) [15].

The problem of control over RPE cell differentiation is of major significance to both biologists and specialists in clinical medicine. In particular, long-standing questions concern the causes of phenotypic changes in the human RPE and ways to regulate fibrotic changes in certain pathological states. A promising way to find the answers is to use well-characterized cell models, provided reliable protocols for effective cell isolation and culturing are available.

## 2. Sources of RPE Cells for Culturing

There are two main sources of RPE cells for model* in vitro* experiments: primary cells and continuous cell lines obtained as a result of spontaneous transformation and immortalization of cells.

### 2.1. Primary Cells

In countries where eye banks are maintained, specialists usually make use of human RPE cells either isolated directly from the initial material (as a rule, cadaver eyes) or available from certain research laboratories. Thus, ScienCell Research Laboratories (USA) offers primary RPE cells (HRPEpiC) isolated from normal human retina and cryopreserved at passage 1 (http://www.sciencellonline.com/), and Lonza Walkersville Inc. (USA) offers Clonetics human primary RPE cells (H-RPE) cryopreserved at passage 2 (http://www.lonza.com).

In countries where no human eye banks exist, primary RPE cells are obtained from the eyes of cows, pigs, rabbits, rats, and other animals [16–19].

Researchers in different laboratories use essentially the same procedure to isolate RPE cells from an adult human eye. The eyeball is cut along the perimeter about 6 mm posterior to the corneal limbus, and its anterior part is discarded [20]. The posterior part is turned upside down to dislodge the vitreous together with the neural retina, and the remains of the retina are then cut off at the optic disc. The resulting cup-shaped segment with RPE on the inner surface is filled with a cell dissociation reagent and incubated at 37°C or room temperature for 8 min to 1 hour. Suitable dissociation reagents include solutions of pronase, papain, trypsin, hialuronidase/collagenase, or dispase [20–24] or of nonenzymatic substances such as EDTA [25, 26]. The solutions are usually prepared in calcium- and magnesium-free Hank's balanced salt solution (HBSS), and the incubation regime depends on the reagent used. The dissociated fragments of RPE are collected with a pipette, pelleted by centrifugation, and resuspended in a complete medium.

To isolate RPE cells from a fetal human eye, the eyeball is cut about 1-2 mm posterior to the corneal limbus to remove the anterior segment, vitreous, and retina [27, 28]. The posterior segment is transferred to a Petri dish with silicone coating and dissected into four quadrants, which are then incubated in dispase solution at 37°C for 30 min. After dispase treatment, sheets of RPE cells are peeled off with forceps under a microscope and collected in tubes with a complete medium [27, 28].

Unlike continuous cell lines, primary RPE cells are relatively heterogeneous, exhibit donor-to-donor variability, and can be expanded for a limited number of passages. Rawes et al. [29] reported that a subculture of adult RPE cells reached replicative failure after 15 population doublings. It is known that aging cells cease to divide, which is explained by alterations in gene expression [30].

### 2.2. Continuous Cell Lines

To date, a variety of continuous RPE cell lines have been produced. They include both human lines listed in Table 1 and, for example, rat cell line RPE-J, which are available from biotechnological companies, in particular, the American type culture collection (ATCC). A major advantage of such lines is that they can be subcultured over more than hundred of passages. Another important feature is that they have a uniform cell composition, although this may be evidence that these lines have lost certain properties essential to the initial cell material.

## 3. Properties of Cell Lines

### 3.1. H80HrPE-6

This dedifferentiated RPE cell line, created by Eguchi et al. from the eye of an 80-year-old man, may form lentoid structures expressing crystallins [22, 32]. This cell line may be a useful system for investigating the regeneration of the lens by human RPE cells [39].

### 3.2. ARPE-19

During the past decade, the ARPE-19 cell line has become most popular in RPE cell research. It has a visually normal karyotype and expresses RPE-specific markers, the retinal pigment epithelium-specific 65 kDa protein (RPE65) and cellular retinaldehyde-binding protein (CRALBP), as has been shown at the mRNA and protein levels, respectively [33]. The properties of ARPE-19 cells depend on culture conditions and the way the cells are maintained and subcultured [40]. Thus, original ARPE-19 cells at passages 15 to 20 in tissue culture flasks produce a uniform epithelial monolayer with typical cobblestone morphology [33], but ARPE-19 strains upon further subculturing change into a heterogeneous mixture of elongate and polygonal cells [40]. In long-term culture on Transwell membranes, ARPE-19 cells have been shown to form a polarized monolayer (see below). ARPE-19 cells have been widely used in studies on oxidative stress, retinal pathogenesis, and signaling pathways and also in research related to drug and toxicity testing [10, 31, 41, 42].

### 3.3. D407

This cell line has typical features of RPE, including cobblestone morphology, phagocytosis of photoreceptor outer segments, and expression of CRALBP protein and cytokeratins (8 and 18) characteristic of RPE [34]. The D407 cells at early passages have a modal chromosome number of 44 ± 2, but by passage 52 they may become almost triploid (71 ± 4 chromosomes) [34]. Unfortunately, D407 cells do not polarize in filter culture and do not synthesize pigment [31, 43].

### 3.4. RPE-340

These cells originally have epithelial morphology in culture, but their replicative ability upon serial passages is limited, and they senesce after 50–60 population doublings by assuming two doublings per passage [35]. RPE-340 cells transfected with hTERT have an extended life span [30, 36].

### 3.5. hTERT RPE-1

This is a near-diploid cell line of female origin with a modal chromosome number of 46 in 90% of the cells counted (http://www.atcc.org/). hTERT RPE-1 cells have been used in studies on the inactive X chromosome (Xi), which provides an excellent model of epigenetic regulation [44, 45].

### 3.6. h1RPE (-7 and -116)

These cells have epithelial morphology with apical microvilli but fail to develop transepithelial electrical resistance (TER) above 30–40 Ω·cm^2^ under normal culture conditions. This cell line has been used in few studies. For example, subretinal transplantation of hRPE cells in Royal College of Surgeons (RCS) rats proved to result in photoreceptors rescue for 5 months after grafting [37]. These cells were found to express P-glycoprotein, but its activity could not be detected [46].

## 4. Cell Culture Conditions for RPE Cells 

Cell differentiation in culture depends on a number of factors, including the composition of the medium and growth substrate. A variety of culture conditions have been used in studies on RPE cells.

### 4.1. Growth Media

The range of media used in RPE cultures includes Iscove's modified Dulbecco's medium (IMDM) [47, 48], Chee's essential medium (CEM) [49], alpha modified Eagle's medium (MEM) [27, 43], Dulbecco's modified eagle medium (DMEM) high glucose [43], and DMEM/F12 [33, 42, 43]. For example, the base media for D407 and ARPE-19 cell lines are DMEM high glucose and DMEM/F12, respectively. Moreover, different supplements to basic media are used to improve the growth and other properties of RPE cells. The proportion of serum added to the base medium varies from 1 to 20%. Chang et al. [17] consider that serum contains a factor that inhibits the formation of tight junctions. The effects of some media supplements on the improvement of barrier properties of the ARPE-19 cell monolayer are described in detail in the study by Mannermaa [31]. Typical supplements used in primary RPE cell cultures include basic fibroblast growth factor (bFGF) and optimized commercial mixtures such as N1 Supplement (containing transferrin, insulin, putrescine, progesterone, selenium, and biotin) or N2 Supplement (based on N1 Supplement without biotin) [25, 26, 28, 49]. Other supplements and different glucose concentrations have also been tested, but there still are no systematic data or any definitive conclusions on the role of media supplements in the properness of cultured RPE-derived cells [31].

### 4.2. Growth Substrate

It has been shown that the presence of the basement membrane is essential for the polarization of RPE cells. Since Bruch's membrane contains laminin and collagens, specialists have widely used growth matrices with these proteins [17, 31]. The same is also true of various biological membranes, such as the amniotic membrane [19].

## 5. Advantages and Disadvantages of Cell Culturing 

The culturing of human RPE cells provides the possibility to analyze in detail their morphology, functions, and molecular and genomic properties under normal and pathological conditions, which is hardly possible* in vivo. *On the other hand, cell culture, as any artificial system, obviously has certain disadvantages [50]. Unlike cells* in vivo*, cultured cells are devoid of their native 3D microenvironment. RPE cells* in vitro* may activate the cell cycle, alter differentiation and behavior, senesce, and undergo apoptosis [7, 14, 51], with culture conditions and certain media components having an effect on their differentiation and viability [27, 52, 53]. Additional limitations on the use of RPE cell cultures arise due to genetic instability of continuous cell lines, which results from their unstable aneuploid chromosome constitution, and heterogeneity of short-term cultures in terms of growth rate and capacity for intrapopulation differentiation, with consequent variation in their properties between passages [54]. Despite all these circumstances, however, cultured cells retain many specialized functions, and cell lines have become an important tool in studies on RPE. The advantage of cell lines is that they maintain their characteristics over a number of passages and have longer survival times, compared to primary cultures. Moreover, many cell lines are homogeneous to a large extent, while primary cultures exhibit heterogeneity and individual donor variability [55]. A noteworthy fact is that RPE cells are not uniform even* in situ, *forming a heterogeneous mosaic of similar but not identical cells [56]. It is important to take into account these features of cultured RPE cells. Anyway, there is no alternative to this approach in studies on cell behavior and molecular mechanisms underlying pathological processes. Currently, both sources of RPE cells for model* in vitro* experiments—primary cells and continuous cell lines—are used in fundamental and applied research, including the development of new approaches to treatment of ophthalmological disorders.

## 6. Approaches to Human RPE Cell Culturing 

Depending on research purposes, human RPE cells are cultured so as to obtain either a highly polarized, functional monolayer of differentiated cells or an adhesive monolayer of dedifferentiated cells on a solid substrate.

### 6.1. Human RPE Cell Culturing under Conditions of a Highly Polarized, Functional Monolayer

This approach is aimed at producing a culture of RPE cells with properties characteristic of the native tissue, including morphological features (apical microvilli, basal invaginations, well-defined tight junctions, and prominent melanocytic pigmentation), expression of specific proteins (CRALBP, RPE65, MITF, Otx2, ZO-1, occludin, claudin, ezrin, Na^+^/K^+^-ATPase, bestrophin, and cytokeratins 8/18), and physiological parameters, TER in particular. To this end, it is expedient to use special culture inserts (e.g., Transwell permeable supports) with a membrane coated with a certain component of extracellular matrix (ECM). The RPE cells are plated onto the membrane at a high density (e.g., 1 × 10^5^ cells per 12 mm diameter insert [28] or 3 × 10^5^ cells per 24 mm diameter insert [57]) and cultured to form a confluent monolayer, which takes 30–60 days. It is only in such a monolayer that epithelial cells produce tight junctions, which provide for a high electrical resistance between electrodes placed in the inner and outer chambers. Cultures derived from the RPE of fetuses at weeks 16–22 of gestation make it possible to obtain a polarized RPE cell monolayer with a high TER (over 500 Ω·cm^2^) [27, 28, 49]. For comparison, this parameter in the human RPE* in vivo* is only 150 Ω·cm^2^ [28].

Polarized monolayer cultures are used as a model for analyzing properties and functions characteristic of native RPE. Moreover, since disturbances of RPE polarization play a major role in the pathogenesis of various retinal diseases, simulation of RPE dysfunction in such cultures provides the possibility to evaluate the ability of RPE to recover under pathological conditions and to test* in vitro *the effects of new medicines. In particular, RPE cells cultured in Transwell inserts are highly suitable for analyzing the transport of various substances and their distribution relative to the insert membrane; polarized secretion of growth factors [33, 48], cytokines [47], and retinoids [58]; and drug toxicity [42].

A polarized functional monolayer can be grown not only from fetal cells but also from human ARPE-19 cell line [33, 43, 47, 59]. For example, Dunn et al. [33] confirmed the ability of ARPE-19 cell line to form polarized monolayers and evaluated their properties, showing, in particular, that FGF5 is secreted from the basolateral surface of ARPE-19 cells. Unlike fetal cells, adult RPE cell lines usually form polarized monolayers with a low TER (<50 Ω·cm^2^) [33, 59]. However, Fragoso et al. [42] managed to obtain ARPE-19 cell monolayers with a TER of about 150 Ω·cm^2^. This is evidence that optimization of protocols for obtaining polarized human RPE cultures from an adult donor is a difficult task that nevertheless should be attempted in view of the special clinical significance of such cultures.

Polarized human RPE cultures derived from donors aged 9–24 years have been used to study polarized secretion of interleukins IL-6 and IL-8 [47], vascular endothelial growth factor (VEGF), and pigment epithelium-derived factor (PEDF) [48, 60]. The level of TER recorded in these cultures is similar to that in monolayers formed by continuous cell lines. The main difficulty in experiments with cultures of adult human RPE is that there is a high probability of change in the morphology of these cells in the course of culturing. Compared to fetal cells, RPE cells from an adult donor are less capable of proper assembly/disassembly of cytoskeleton and cell-to-cell contacts in the course of proliferation, which may eventually result in EMT. However, Blenkinsop et al. [61] have developed an optimized protocol where the growth medium is supplemented with several factors used for culturing fetal RPE cells (taurine, hydrocortisone, and triiodothyronine), which makes it possible to obtain a functional monolayer of adult RPE cells with a TER of about 200 Ω·cm^2^.

### 6.2. RPE Cell Culturing as an Adhesive Monolayer on a Solid Substrate

This approach has been used in the majority of studies on phenotypic changes in RPE cells evaluated by morphological and molecular genetics methods. It has been shown that RPE cells in such cultures gradually lose polarity and specialized cell-to-cell contacts characteristic of epithelia and acquire certain features of mesenchymal cells, including migration behavior [15]. Similar changes take place* in vivo *during EMT in the embryonic neuroectoderm, with RPE being one of its derivatives. In particular, cells from the roof plate of the neural tube undergo EMT and delaminate from the neuroepithelium to form a migratory population of multipotent mesenchyme-like neural crest cells [62, 63].

One of the early events in EMT is the disassembly of tight junctions, with consequent redistribution of zonula occludens (ZO) proteins, claudins, and occludin, the disruption of the polarity complex, and the initiation of cytoskeletal reorganization [64]. Experiments with the ARPE-19 cell line have shown that these cells gradually lose tight junctions but continue to express ZO-1 and occludin proteins [40]. In primary cultures of human RPE cells and in rat RPE-J cell line, the loss of polarity in the expression of Na^+^/K^+^-ATPase has been observed [65], with this enzyme being revealed not only on the apical but also on the basolateral surface of RPE cells [66].

Subsequent EMT events include disassembly of adhesion contacts and reorganization of the polarized epithelial actin cytoskeleton into actin stress fibers anchored to the focal adhesion complexes [64]. The attachment of actin microfilaments to the cytoplasmic membrane in cultured human RPE cells is facilitated due to the synthesis of vinculin, which contributes to the binding of cell surface integrin receptors to ECM adhesion molecules [67].

A basic factor of cytoskeletal reorganization is the cessation or reduction of E-cadherin expression, which is also observed in RPE cells* in vitro.* This is accompanied by increased expression of N-cadherin, a marker of neural cell contacts [25, 68–70], which is evidence for the loss of epithelial cell organization [71]. As a result of aforementioned rearrangements, the cells undergoing EMT acquire a mesenchyme-like phenotype characterized by the expression of corresponding cytoskeletal proteins (namely, vimentin) and increased deposition of ECM proteins, including collagen and fibronectin [62, 72]. All these events also take place in RPE cells grown in culture flasks. Thus, human RPE cells grown* in vitro* show distinct positive staining for vimentin [73] and synthesize various ECM molecules such as tissue inhibitor of metalloproteinase 3 (TIMP-3) [74]; collagen types I [26], IV, and V [75]; laminin [76]; fibronectin [26, 77, 78]; heparan sulfate proteoglycan; and hyaluronic acid [79, 80].

The secreted ECM components (collagen and fibronectin) stimulate integrin signaling and consequent formation of focal adhesion complexes, which facilitate cell migration [72]. This fact has also been confirmed for RPE cells. Adult human RPE cells cultured* in vitro, *compared to native RPE, show an increased expression of integrins, which form receptors for laminin, fibronectin, and collagen, thereby making the attachment of cells to the substrate more effective and facilitating their migration [77]. The secretion of ECM proteins by RPE cells can also be stimulated by certain factors added to the culture medium. For example, protein S100*β* stimulates fibronectin secretion [41, 81], and TGF-*β*1 added to ARPE-19 cell culture enhances the expression of fibronectin, laminin, matrix metalloproteinase 2 (MMP-2), and collagen type I [10, 82].

Dedifferentiation of adult human (or animal) RPE cells* in vitro *is accompanied by the onset or intensification of expression of proteins associated with motor cell function. Thus, *α*-smooth muscle actin (*α*-SMA), a marker of myogenesis, appears in cells that acquire a spindle-shaped morphology [10, 82]. However, neither the *α*-actinin-1 isoform specific for skeletal muscle cells [55] nor markers of mesenchymal stem cells such as STRO-1 [25, 55], CD90, and CD105 [55] can be detected in human RPE cell culture.

The expression of desmoplakin and other desmosomal components decreases in the course of EMT, but the effect of this decrease on other events involved in EMT is as yet unclear. It has only been shown that changes take place in the expression pattern of cytoskeletal proteins, including a decline in the expression of specific intermediate filaments structurally associated with desmosomal proteins. RPE cells cultured* in vitro* cease to express cytokeratins 8 and 18, which are characteristic of native RPE [83, 84] but start to express cytokeratins 7 and 19 [67, 84]. There is evidence that cytokeratin 19 is synthesized in migrating RPE cells [84].

Thus, RPE cells cultured as an adhesive monolayer gradually lose epithelial characteristics, including polarity and specific markers (pigmentation and expression of E-cadherin, CRALBP, and cytokeratins 8 and 18) and acquire migratory properties and mesenchymal cell-like features (e.g., express collagen type I and fibronectin), which is similar to phenotypic changes of RPE cells* in vivo* under pathological conditions.

In addition to phenotypic manifestations of EMT, RPE cells* in vitro *begin to display some features characteristic of neural cells, which may reflect the neuroepithelial origin of the RPE itself. Thus, adult rat RPE cells in culture were shown to express both neuronal markers—nestin, *β*-tubulin 3 (TUBB3), cortin, NG2, MAP2, and 200 kDa neurofilament protein (neurofilament 200)—and glial cell marker (GFAP) [85]. Using immunohistochemical methods and Western blot analysis, Vinores et al. [86] found that TUBB3 (an early neuronal marker) was not initially expressed in a primary culture of adult human RPE but could be detected beginning from day 5, with its expression being maintained in subsequent monolayer subcultures. Experiments with human RPE cell lines H80HrPE and ARPE-19 confirmed that these cells were immunoreactive for TUBB3 and could be induced to express mature neuronal protein markers NSE, MAP5, and neurofilament 200 [7].

**Figure 1:**
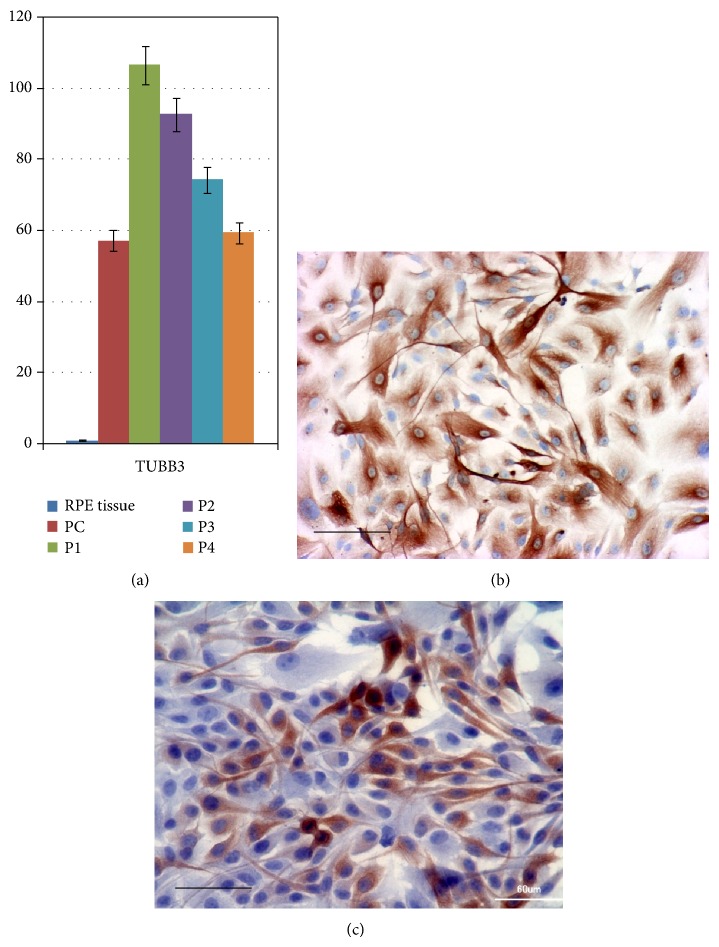
Some characteristics of adult human RPE cells* in vitro*. (a) The results of real-time PCR analysis of* TUBB3* expression in primary culture (PC) and subsequent passages (P1–P4) of RPE cells in adhesive monolayers, compared to freshly isolated RPE (RPE Tissue). (b) Immunoperoxidase staining for TUBB3 (brown) in passage 2 RPE cells. (c) Immunoperoxidase staining (brown) for neurofilaments 68 and 200 in passage 4 RPE cells. Cell nuclei are stained with hematoxylin. Scale bar, 60 *μ*m.

Our immunohistochemical and molecular genetic studies on primary cultures of adult human RPE cells have shown that they begin to express stem cell gene markers such as* Oct4 (POU5F1), Nanog, Prox1, Musashi 1, *and* Pax6*, which is evidence for dedifferentiation of RPE cells in the course of culturing [87]. Moreover, these cells are capable of subsequent transdifferentiation into neural cells, as indicated by the expression of* Musashi 1, Pax6, *and* TUBB3 *(Figure 1(a)) and positive staining with antibodies against protein markers of neuronal differentiation—nestin, TUBB3 (Figure 1(b)), tyrosine hydroxylase, neurofilaments 68 and 200 (Figure 1(c)), and nNOS—and glial differentiation (CNPase, GFAP) [25, 26, 87, 88]. RPE cells* in vitro* also show positive staining for vimentin, a marker of intermediate filaments [26, 73]. Simultaneous expression of vimentin and intermediate filament proteins of other classes, nestin and GFAP, observed in RPE cell culture [26] is also characteristic of human neural stem cells [89]. This fact indicates that RPE cells* in vitro* are apparently multipotent.

Evidence for multipotency of adult human RPE cells has also been obtained by other authors. Thus, Salero et al. [55] have shown that a subpopulation of these cells* in vitro *can be activated into self-renewing retinal pigment epithelial stem cells (RPESCs) that lose RPE markers, proliferate, and, depending on culture conditions, can either redifferentiate into stable RPE monolayers or transdifferentiate into neural or mesenchymal cells (adipocytes, chondrocytes, or osteogenic cells). In other words, RPESCs are multipotent stem cells that, under certain conditions, can generate both neural and mesenchymal progeny.

Experimental evidence that adult human RPE cells* in vitro* can acquire some features of neural cells suggests the existence of factors preventing RPE transdifferentiation into neural retinal cells* in situ*. Therefore, the search for means to induce RPE cell differentiation into neuronal direction, suppressing their mesenchymal differentiation, is of obvious fundamental and practical importance and may offer new possibilities for restoring the retina after injury or pathology.

## 7. Role of Signaling Pathways in Phenotypic Changes of RPE Cells* In Vitro*

Morphological and functional changes in RPE cells cultured as adhesive monolayers are similar to those observed in the RPE of patients with various degenerative or proliferative vitreoretinal diseases. For this reason, such cultures are used as* in vitro *model systems to study factors responsible for changes in RPE cells (with regard to their proliferation, migration, and differentiation) and signaling pathways coordinating the mechanisms of cell-to-cell interactions in the course of these processes.

Cells need to sense cues from their extracellular environment and integrate this information into appropriate developmental or physiological responses. Although there are a number of mechanisms that relay information from the exterior to the interior of the cell, a relatively small set of highly evolutionarily conserved signaling pathways stand out as playing particularly important roles in this transmission of information [90]. In particular, they include Shh, Wnt, Notch, TGF-*β*/BMP, EGFR, PI3K/AKT/mTOR, JAK/STAT, and nuclear hormone receptor (NHR) pathways [90, 91]. Each of the pathways converts information about the concentration of extracellular ligands into specific transcriptional responses in the cell nucleus.

### 7.1. TGF-*β*/BMP Signaling Pathway

The TGF-*β* superfamily of ligands in mammals comprises not only three isoforms of this factor (TGF-*β*1, TGF-*β*2, and TGF-*β*3) but also other signaling proteins of similar structure, such as bone morphogenetic proteins (BMPs), growth and differentiation factors (GDFs), activins, and inhibins [92].

**Figure 2:**
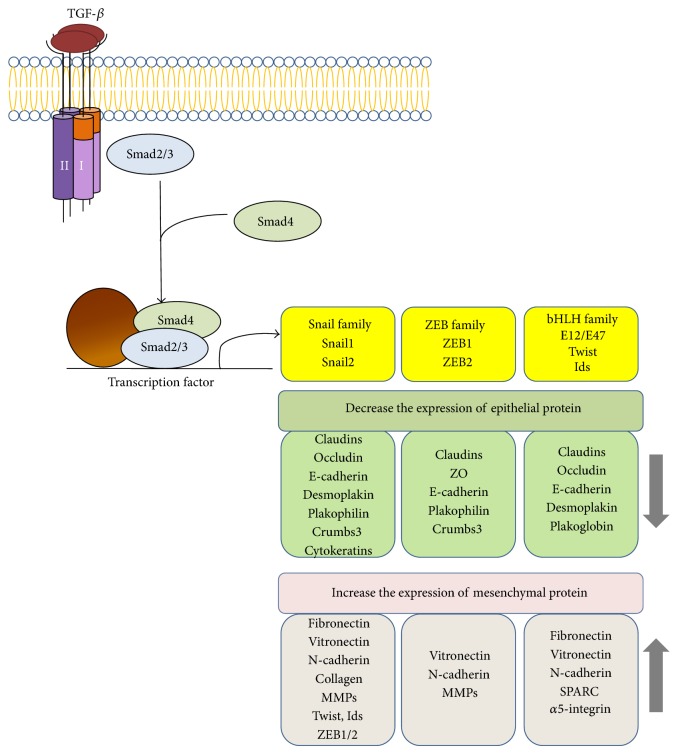
Transcriptional regulation of EMT induced by TGF-*β* (according to Xu et al. [94]). In response to TGF-*β*, Smad2 and Smad3 are activated to form complexes with Smad4, which then regulate the transcription of target genes through interactions with other DNA binding transcription factors. In the induction of EMT, the activated Smads mediate transcriptional regulation through three families of transcription factors, which results in repression of epithelial marker gene expression and activation of mesenchymal gene expression.

A TGF-*β* ligand binds to a specific type II receptor dimer, which recruits a type I receptor dimer, both of them forming a complex with the ligand. The respective receptors for TGF-*β* ligands are named TGF*β*R2 and TGF*β*R1; for BMPs BMPR2 and BMPR1; and so forth. These are serine/threonine protein kinase receptors, and the type II receptor in the complex catalyzes phosphorylation of the type I receptor, thereby activating the latter. The type I receptor, in turn, phosphorylates receptor-regulated Smad proteins involved in different intracellular pathways: Smad2 and Smad3 in the TGF-*β* pathway or Smad1, Smad5, and Smad8 in the BMP pathway [93, 94]. These phosphorylated proteins form heteromeric complexes with Smad4 (a co-Smad) that enter the nucleus and interact with DNA-bound transcription factors of the Snail, ZEB, and bHLH families, which activate or suppress the transcription of genes involved in EMT (Figure 2).

TGF-*β* induces the expression of connective tissue growth factor (CTGF), and both these factors as strong activators of the synthesis and accumulation of ECM proteins play a key role in the development of PVR and transformation of RPE into fibroblast-like cells* in vitro* [10, 14]. Thus, experiments with ARPE-19 cells have shown that TGF-*β* and CTGF enhance the expression of ECM components such as fibronectin, laminin, MMP-2, and collagen type I; as a result, the cells undergo rearrangements in the cytoskeleton, start to express *α*-SMA, and acquire a mesenchymal phenotype [10, 82]. In the D407 cell line, TGF-*β* and activin A proved to stimulate not only reorganization of the cytoskeleton but also cell migration, acting through the TGF-*β*/Smad signaling pathway [93].

As shown by Li et al. [95], TGF-*β*1 induced EMT in ARPE-19 cells, as followed from the expected decline of E-cadherin and ZO-1 expression and enhancement of fibronectin and *α*-SMA expression, with the associated increase in the expression of Snail transcription factor at both mRNA and protein levels. Snail silencing significantly attenuated TGF-*β*1-induced EMT, reducing the expression of mesenchymal markers (fibronectin and *α*-SMA) and enhancing that of the epithelial marker E-cadherin and ZO-1. Snail knockdown could effectively suppress ARPE-19 cell migration. Finally, Snail was activated in epiretinal membranes from PVR patients. Thus, Snail plays an important role in TGF-*β*-1-induced EMT in human RPE cells and may contribute to the development of PVR, while its specific inhibition may provide a new approach to the prevention and treatment of PVR [95].

The results of numerous experiments with knockout animals show that BMPs (in particular, BMP-4 and BMP-7) play a major role in eye morphogenesis [96–98] and RPE specialization [99, 100], but information on the functions of BMPs and their receptors in the adult RPE under normal or pathological conditions is scarce. Mathura et al. [101] were the first to evaluate the expression of BMP-4 and BMPR2 mRNAs in fresh isolates of adult human RPE cells, their primary cultures, and the ARPE-19 cell line. BMP-4 has been shown to inhibit RPE cell proliferation [101]. As shown in subsequent studies, BMP-4 is differentially expressed in the macular RPE of patients with dry or wet AMD [102, 103], depending on microenvironment [104]. Thus, BMP-4 expression in the dry form is enhanced, but in the wet form it is reduced so that the protein cannot be detected by immunochemical methods in surgically excised choroidal neovascular (CNV) membranes [102] consisting of vascular endothelial cells, macrophages, and transdifferentiated RPE cells [8]. In dry AMD, BMP-4 mediates oxidative stress-induced RPE senescence and is responsible for increased p53 protein contents in RPE cells [103]. Therefore BMP-4 appears to be a new potential therapeutic target for suppressing the effects of oxidative stress and RPE senescence in dry AMD [103]. The data obtained by Xu et al. [104] appear to explain the mechanism of BMP-4 downregulation in CNV. These authors have found that the level of tumor necrosis factor alpha (TNF*α*), a major pleiotropic inflammatory cytokine, inversely correlates with the level of BMP-4 in laser-induced CNV lesions in mice, indicating that TNF*α* inhibits BMP-4 expression in the RPE cells during active CNV development. They have also shown that TNF*α* significantly downregulates BMP-4 expression in cultured human fetal RPE cells, ARPE-19 cells, and RPE cells in murine posterior eye cup explants [104].

Signaling proteins of the TGF-*β* family have a regulatory effect on EMT and can reverse this process during embryonic development and normal wound healing. Moustakas et al. [92] have shown that TGF-*β* acting on polarized epithelial cells stimulates their transformation into mesenchymal cells, while treatment of mesenchymal cells with BMPs stimulates mesenchymal-to-epithelial transition. However, the balance between EMT and reverse transition is thought to become deregulated under pathological conditions such as chronic inflammation, resulting in development of fibrotic disorders. Therefore, agents capable of inhibiting the EMT of RPE cells may be of great therapeutic value in the prevention of PVR after retinal detachment or active CNV development. For this reason, the mechanism of BMP4 downregulation revealed by Xu et al. [104] may be useful for defining novel targets for AMD therapy.

**Figure 3:**
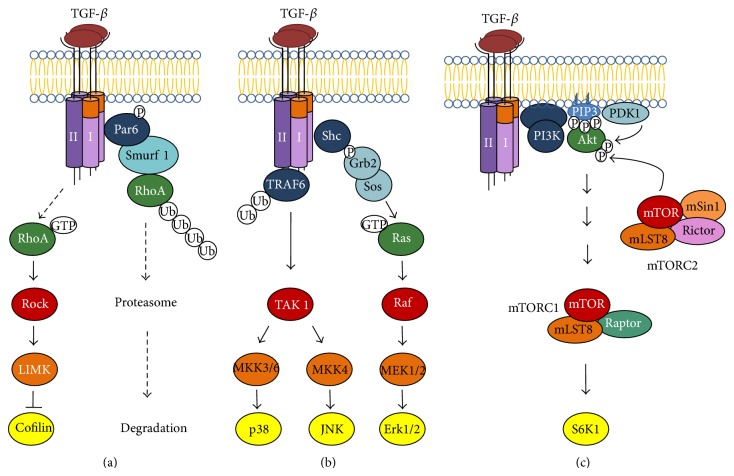
Non-Smad signaling in response to TGF-*β* (according to Xu et al. [94]). (a) Activation of RhoA in response to TGF-*β* and induction of ubiquitin-mediated RhoA degradation at tight junctions. (b) TGF-*β* activates p38 MAP kinase and JNK MAP kinase signaling through the activation of TAK1 (TGF-*β*-activated kinase) by receptor-associated TRAF6, and ERK MAP kinase signaling through recruitment and phosphorylation of Shc by the T*β*R1 receptor. (c) TGF-*β* induces PI3-kinase signaling, leading to the activation of AKT-mTOR signaling and consequently to increased translation.

In addition to the canonical TGF-*β*/Smad pathway, there are also non-Smad-signaling cascades. Recent studies on changes in the RPE cytoskeleton under the effect of TGF-*β*1 confirm that this factor plays a major role in such cascades, in particular, the* Rho*А*/ROCK signaling cascade* (Figure 3(a)) [9, 10, 82]. The RhoA protein, which is important for the formation and maintenance of cell-to-cell contacts [105], is a small GTPase of the Rho family. These GTPases activate Rho-associated protein kinase (ROCK) belonging to the superfamily of serine/threonine protein kinases. About 20 substrates phosphorylated by ROCK are known. They include cytoskeletal proteins, myosin light chains, myosin phosphatase, and LIM kinase, which plays an important role in actin polymerization by phosphorylating cofilin [106]. ROCK is involved in various functions and activities of cells, including organization of the cytoskeleton, formation of stress fibers and focal contacts, proliferation, migration, and apoptosis [107]. Thus, TGF-*β*1 treatment of primary isolates of adult human RPE cells and ARPE-19 cell line resulted not only in the increased phosphorylation of Smad2/3 but also in the RhoA and Rac1 activation [9, 82]. Fibroblast-like changes in the cytoskeleton of ARPE-19 cells could be prevented by cell pretreatment with hydroxyfasudil, a specific inhibitor of Rho [82]. Moreover, the expression of fibronectin, MMP-2, and collagen type I in these cells was blocked when the culture medium was supplemented with Y27632, a specific small-molecule inhibitor of ROCK. This is evidence that the expression of mesenchymal ECM components is enhanced due to activation of the RhoA/ROCK signaling cascade, while suppression of this cascade reduces manifestation of mesenchymal properties in transformed RPE cells.

In the normal eye, TGF-*β* has been revealed in photoreceptors, aqueous humor, hyalocytes of the vitreous body, and choroid [108], and its expression has proved to increase in PVR [82, 109]. Huang et al. [9] cultured early passages of adult human RPE cells (from healthy donors) in the presence of 25% vitreous humor and revealed rearrangements in their cytoskeleton that were similar to those observed by Lee et al. [82] in ARPE-19 cells treated with TGF-*β*1. These rearrangements, however, could be prevented by treating RPE cells with NSC23766, a specific small-molecule inhibitor of Rac1 activation. It is known that the Rac protein plays a key role in the regulation of actin polymerization and contributes to the formation of lamellipodia at the leading edge of migrating cells [9]. Zhu et al. [10] consider that an effective way to prevent RPE cell transformation (EMT) under the effect of TGF-*β* is to inhibit the RhoA/ROCK signaling cascade in these cells. This appears to be a promising therapeutic approach to PVR treatment, which is being developed on the model of RPE cell cultures.

Chung et al. [12] in experiments of mouse RPE cell culture have shown that TGF-*β* activates Ras proteins (Figure 3(b)) involved in different signal transduction cascades, including the well-studied* MAPK/ERK signaling cascade*. Mitogen-activated protein kinases (MAPK) are involved in signal transduction from membrane receptors to transcription factors in the nucleus. They comprise three small protein kinase families: p38 mitogen-activated protein kinases (p38 MAPK), c-Jun N-terminal/stress-activated protein kinases (JNK/SAPK), and extracellular signal-regulated kinases (ERK). Activation of ERK kinases is almost always connected with cell survival, stimulation of proliferation, and activation of p38 and JNK kinases, with induction of apoptosis [110]. The data by Chen et al. [111] provide evidence that ERK1/2 signaling pathway can cross-interact with the canonical TGF-*β*/Smad and the Jagged/Notch pathways in RPE cells during EMT. In particular, these authors have shown that the activation of ERK1/2 signaling by TGF-*β*2 is independent of the canonical TGF-*β*2/Smad pathway in ARPE-19 cells. On the other hand, inactivation of ERK1/2 signaling by U0126, a small-molecule phosphorylation inhibitor of MEK-1/2 (a type of MAPK/ERK kinase), prevents TGF-*β*2-induced downregulation of P-cadherin and upregulation of *α*-SMA, collagen type IV, N-cadherin, and fibronectin in the RPE cells through inhibiting both canonical TGF-*β*2/Smad and Jagged/Notch pathways. Finally, Notch pathway blockade with specific inhibitor DAPT can suppress TGF-*β*2-induced activation of ERK1/2 pathway [111].

According to Chung et al. [12], the Ras-ERK signaling pathway is involved in the regulation of neuronal cell differentiation. Thus, TGF-*β* added to the culture medium of mouse RPE cells proved to enhance the expression of neuron-associated genes,* TUBB3 *in particular; on the other hand, cell pretreatment with U0126 effectively blocked TGF-*β*-induced ERK phosphorylation and markedly suppressed* TUBB3 *expression. These results show that TGF-*β* stimulates* TUBB3* expression by activating the MAPK/ERK signaling pathway and agree with published data on the involvement of the MAPK/ERK pathways in RPE transdifferentiation into the neural retina. In particular, it has been shown that the ectopic expression of a constitutively activated allele of MEK-1 (MEK^*DD*^), the immediate upstream activator of MAPK/ERK, in chicken embryonic retina* in ovo* induces transdifferentiation of the RPE into a neural-like epithelium, which is correlated with downregulation of* MITF *expression in the presumptive RPE [112]. Therefore, TGF-*β* activates RPE cell differentiation in both mesenchymal and neuronal directions.

Saika et al. [113] have reported that p38 MAPK is involved in EMT of RPE cells: inhibition of p38 MAPK by the specific inhibitor, SB202190, interferes with stimulatory effects of exogenous TGF-*β*2 on migration of ARPE-19 cells and on production of ECM components, such as collagen type I and fibronectin.

Further evidence that TGF-*β* activates non-Smad MAPK/ERK and PI3K signaling pathways (Figures 3(b) and 3(c)) comes from the studies by Lee et al. [82] and Huang et al. [9]. They show that the level of ERK1/2 and AKT phosphorylation in human RPE cells increases after treatment with TGF-*β*1, compared to that in control cells. Activation of the* PI3K/AKT/mTOR pathway* by TGF-*β* is of special interest in view of the data by Zhao et al. [114] on its role in RPE dedifferentiation and hypertrophy. These authors experimented on transgenic mice with an RPE-selective postnatal loss of mtDNA transcription and replication in which early activation of this pathway accounted for dedifferentiation of the RPE, with morphological changes in it being similar to those observed in human retinal diseases. They found that RPE dedifferentiation and consequent degeneration of photoreceptors could be prevented by blocking mammalian target of rapamycin (mTOR) activation with rapamycin, an inhibitor of mTORC1 (the intracellular mTOR form sensitive to this inhibitor). Thus, specific inhibition of this pathway, mTOR in particular, appears to be a valid strategy for the treatment of degenerative retinal diseases caused by RPE damage.

**Figure 4:**
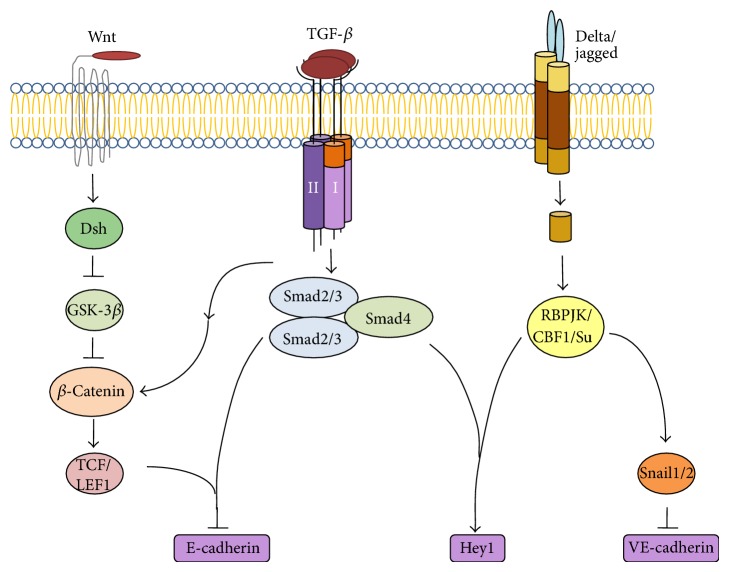
Signaling crosstalk between the TGF-*β*-activated pathway and other pathways during EMT (according to Xu et al. [94]).

Thus, the above data on the involvement of TGF-*β* in the activation of RhoA/ROCK and MAPK/ERK signaling pathways in the RPE suggest that postnatal RPE cells cultured* in vitro* not only undergo EMT but, in parallel, also transdifferentiate into neural cells, but this transdifferentiation in higher vertebrates, including humans, is not completed. To reveal factors restraining neural transdifferentiation of human RPE cells and understand the mechanisms of signaling responsible for EMT, it is necessary to take into account the crosstalk between the TGF-*β*/BMP and other signaling pathways, including the Wnt and Notch cascades (Figure 4).

Although TGF-*β* appears to play a key role in stimulating RPE cells to form a PVR membrane, many other factors may be involved in pathogenesis of vitreoretinal disorders and other EMT-related retinochoroidal diseases. They include platelet derived growth factor (PDGF) [115, 116], heparin-binding epidermal growth factor (HB-EGF) [117, 118], hepatocyte growth factor (HGF) [118], epidermal growth factor (EGF) [118], and TNF*α* [119–121]. It has been shown that the contents of various growth factors and cytokines, which are inflammatory products of cell activation, are increased in vitreous aspirates from the eyes with PVR [118, 119].

According to Liu et al. [120], TNF*α* activates AKT, mTORC1, and mTORC2 signaling in cultured ARPE-19 cells; however, it is AKT/mTORC1, but not mTORC2, signaling that is required for TNF*α*-mediated RPE cell migration* in vitro*. As shown in their subsequent study, mTORC1 (but not mTORC2) signaling is important for matrix metalloproteinase 9 (MMP-9) expression in RPE cells [121].

Takahashi et al. [119] have shown that TNF*α* induces the formation of fibrotic foci by cultured ARPE19 cells through activation of TGF-*β* signaling in a manner dependent on hyaluronic acid-CD44-moesin interaction. TNF*α* promotes the expression of CD44, the principal transmembrane adhesion receptor for hyaluronic acid, and moesin phosphorylation by protein kinase C (PKC), which leads to the pericellular interaction of hyaluronic acid and CD44. The formation of the hyaluronic acid-CD44-moesin complex results in cell-cell dissociation and increased cellular motility through actin remodeling. Furthermore, this complex has proved to associate with TGF*β*R2 and clathrin at actin microdomains, with consequent activation of TGF-*β* signaling and induction of the mesenchymal phenotype in RPE cells. Furthermore, the authors have demonstrated that the development of fibrosis induced by injection of TNF*α* into the mouse retina is markedly suppressed in CD44 knock-out mice. These findings indicate that the hyaluronic acid-CD44 interaction plays a key role in EMT-associated fibrotic disorders.

Chen et al. [118] have reported that HGF coupled with EGF or HB-EGF induces migration of both primary RPE cells and ARPE-19 cells in a synergistic manner, via enhancement of PKC*δ* and ERK.

### 7.2. EGFR Signaling Pathway

The actions of EGF, including those related to cell survival, begin with the binding of this factor to its receptor (EGFR), which belongs to the ErbB family of receptor tyrosine kinases. The interaction of EGF or any other specific ligand (e.g., TNF*α*, HB-EGF, and betacellulin) with EGFR (ErbB1) induces receptor dimerization, which activates an intrinsic tyrosine-specific kinase [122]. It has been shown that EGF enhances the survival of RPE D407 cells in serum-free suspension culture via signaling through both PI3K and ERK/MAPK pathways, with this effect of EGF being substantially reduced by either the PI3K inhibitor LY294002 or the MEK1/2 inhibitor U0126 [122].

As follows from the above data, different growth factors exert their effects on the cell via different signaling pathways, and this should be taken into account when developing drug therapy against EMT of RPE cells in fibrotic disorders. Consideration should also be given to crosstalk between different signaling pathways. For example, if phosphorylation of AKT and ERK1/2 in RPE cells is blocked in order to inhibit EMT, then the protective effect of EGF or other such factors on cell survival will also be blocked.

### 7.3. Wnt/*β*-Catenin Signaling Pathway

The canonical Wnt/*β*-catenin signaling pathway plays a key role in the regulation of tissue differentiation not only in the course of embryonic development but also in the postnatal period, having an effect on cell proliferation, senescence, and tumor growth [123]. *β*-Catenin is the central mediator of this pathway. For example, it accounts for activation of* MITF и TYR* genes in the committed Otx2^+^ precursor cells of the developing retina, which leads to their differentiation into RPE cells [124].

When the Wnt pathway in the RPE is inactive, *β*-catenin is contained in the cytoplasm and cell membranes, where it is phosphorylated and associated with E-cadherin. The association between these proteins is indicative of stable cell-to-cell adhesion. This pathway is activated by extracellular Wnt ligands, which interact with Frizzled receptors and their Lrp5/6 coreceptors on the cell membrane and thereby inhibit *β*-catenin phosphorylation. As a result, its binding to E-cadherin is hindered, with consequent impairment of cell adhesion, and *β*-catenin is translocated to the nucleus [125]. In the RPE cell nuclei, *β*-catenin interacts with T-cell-specific transcription factor (TCF) to form the *β*-catenin-TCF complex which induces gene transcription, including that of* cyclin D1* and* c-Myc* [123, 126, 127]. This leads to activation of cyclin-dependent kinases responsible for cell cycle progression through G1 to S phase.

In the postnatal period, Wnt/*β*-catenin signaling in RPE cells regulates the expression of genes pertaining to the antioxidant protection system [42, 128]. A protective effect of the Wnt3a ligand was demonstrated in experiments on ARPE-19 cells treated with Wnt3a in the presence or absence of cytotoxic agents, hydrogen peroxide, and paraquat. The results showed that such treatment improved cell viability, with its effect being mediated by STAT3 activation [42].

Rak et al. [129] in calcium switch experiments on adult human RPE cell cultures revealed a relationship between calcium-dependent cell adhesion, morphology, and pigmentation. The observed changes in cell morphology (gradual transformation of pigmented cells with an epithelial phenotype into spindle-shaped depigmented cells) proved to be reversible, depending on calcium concentration in the medium. The RPE cells were plated at high density in a low-calcium medium and cultured through at least six serial passages to minimize their differentiated properties. Thereafter, they were transferred to a high-calcium medium and maintained at confluence for up to 4 months, being examined for phenotype, pigmentation, and the expression of epithelial cell markers by Western blot analysis. The calcium switch resulted in a rapid restriction of N-cadherin to lateral cell borders and expression of tyrosinase by day 4. The pigment was again detected in the cells after 8 weeks; CRALBP expression, after 12 weeks; and myocilin, after 4 months. Myocilin is known to have a role in actin cytoskeletal reorganization, cell-to-cell interactions, and cell migration. This protein is a modulator of the Wnt cascade: it competes with Wnt for binding with certain Frizzled receptors and interacts with *β*-catenin [130].

Mechanisms of Wnt/*β*-catenin signaling can provide explanation for many pathological processes associated with changes in the structure and function of RPE cells. Studies in this field are developing rapidly, since disclosure of these mechanisms and approaches to their regulation will help in understanding the essence of EMT not only of RPE cells* in vivo* but also of pathological processes in the senescent RPE.

### 7.4. JAK/STAT Signaling Pathway

Signal transducers and activators of transcription (STATs) comprise a well-characterized family of proteins that transmit a signal from the cell surface to the nucleus and directly participate in gene regulation and cell responses to cytokines and growth factors. In particular, the STAT3 protein induces the expression of antiapoptotic genes in various tissues and activates receptors for IL-6, leukemia inducing factor (LIF), ciliary neurotrophic factor (CNTF), and tyrosine kinase. STAT3 is expressed in the developing and adult RPE and neural retina [131]. Its expression in the RPE of patients with AMD increases upon formation of CNV membranes [132]. In the ARPE-19 cell line, STAT3 activation has proved to result in enhancement of proliferation. JAK/STAT signaling can initiate angiogenesis by activating the production of angiogenic factors, including VEGF and MMPs. Analyzing cytokine-induced changes in the JAK/STAT pathway on the model of ARPE-19 cells, Fasler-Kan et al. [132] have shown that different cytokines (interferon-*α*, interferon-*γ*, IL-4, and IL-6) are involved in stimulation of different signaling molecules (STAT1, STAT2, STAT3, or STAT6). As STAT3 plays a central regulatory role in the pathogenesis of AMD, specialists regard it as a potential therapeutic target for the treatment of this disease.

Fragoso et al. [42] in experiments with ARPE-19 cells were the first to reveal the relationship between Wnt3a -mediated STAT3 activation and cell survival, showing that there is a crosstalk between the corresponding two signaling pathways. The role of STAT3 in the Wnt pathways is so significant that STAT3 knockdown by siRNA impairs Wnt3a-dependent cell protection from oxidative stress.

### 7.5. Notch Signaling Pathway

In canonical Notch signaling, a Notch transmembrane receptor undergoes proteolysis in a presenilin/*γ*-secretase-dependent manner when exposed to ligand-expressing cells (Delta or Serrate/Jagged). Proteolysis of the Notch receptor releases the Notch intracellular domain (NICD), which translocates to the nucleus, where it binds to the transcription factor RBP-Jk/CBF1/Su(H) and converts it from a repressor into an activator of target genes, including the Hes and Hey family genes [90, 94]. In epithelial cells, for example,* Hey1* was found to be required for TGF-*β*-induced EMT and migration [94].

Studies on the role of Notch signaling pathway in pigment cells began relatively recently. It has been shown that Notch signaling, mediated in rodents by the RBP-Jk transcription factor (homologous to human CBF1), is necessary for self-maintenance of melanoblasts and melanocyte stem cells [133]. Available data on its role in the regulation of RPE differentiation have been obtained only in experiments with animal models. During normal development, RPE cell differentiation is regulated via the canonical Notch signaling pathway [134], with its target gene* Hes1* being implicated in the formation of the lens, optic cup, and RPE in early embryos [135]. Optic cup and lens defects, plus precocious neurons, were found in E10.5 Hes1^−/−^ eyes [136]. Loss- and gain-of-function studies in the late embryonic and postnatal mouse retina demonstrate that* Hes1 *represses the formation of retinal ganglion cells, rods, and horizontal and amacrine neurons [136, 137]. Lee et al. [135] propose that* Hes1* is a temporal brake that integrates the timing of neurogenesis with morphogenesis. According to recent data, constitutive activation of RBP-Jk-dependent Notch signaling during mouse embryonic development leads to hyperproliferation and tumor formation in the adult RPE [138].

Experiments of Saad et al. [139] with kidney tubule epithelial cells have shown that Notch signaling combined with Snail expression plays an important role in EMT and fibrosis formation. Notch inhibition by DAPT in the course of EMT has proved to retard decrease in E-cadherin expression and increase in *α*-SMA, MMP-2, and MMP-9 expression, with the level of Snail expression being also reduced. The authors consider that inhibition not only of Snail but also of Notch can provide a means of control over EMT.

As noted previously, Notch signaling can cross-interact with both canonical Smad-dependent and noncanonical TGF-*β* signaling pathways in RPE cells during EMT [111]. Moreover, it has also been shown that elements of the Notch signaling pathway—including Jagged-1, Notch-3, Hes-1, and Hey-1—are upregulated in TGF-*β*2-stimulated EMT in human RPE cells, while blockade of this pathway with DAPT completely reverses TGF-*β*2-induced EMT [140].

Not only does the Notch signaling pathway interact with Wnt and TGF-*β*/BMP but there is also evidence for its interactions with other pathways, such as Shh and NF-*κ*B [141], but their role in phenotypic changes of human RPE cells* in vitro* has not yet been studied.

## 8. Prospective Therapeutic Agents 

Several strategies aimed at the inhibition of signaling pathways involved in RPE pathologies have been elaborated to date [142, 143]. Modulation of signal transduction molecules—for example, RhoA/Rho-kinase, Smad, or MAPK—by small molecules, gene transfer, or some other technology appears promising as a means of prevention and treatment of such pathologies [143]. Systemic administration of ALK5 inhibitors effectively suppresses fibrogenic reaction and development of tissue fibrosis in animals [144–146]. Increasing attention has been recently devoted to the inhibitory role of different microRNAs [147], specific small molecules [148–151], antibiotics, immunosuppressants [114], steroids [152], and histone deacetylase inhibitor [153]. Another group of interest comprises antiangiogenic agents capable of blocking different steps in the pathway of angiogenesis under pathological conditions: antibodies to the VEGF, novel steroids, triamcinolone acetonide, siRNAs, high-affinity VEGF antagonists (angiostatin, endostatin), PEDF, and so forth, [154]. Future studies are needed to identify other key modulators involved in the process of RPE damage, which is necessary for gaining a deeper insight into the causative mechanisms of RPE pathologies and finding effective ways of their prevention and treatment.

## 9. Conclusions

Human RPE cell cultures provide wide possibilities for research on the mechanisms of pathological processes taking place* in vivo* and the methods of their regulation at the cell and molecular levels. Depending on culture conditions, RPE cells can change their differentiation status, losing cell type-specific features and redifferentiating into epithelial cells. In directed experiments, adult RPE cells undergo EMT and acquire certain properties of mesenchymal and proneural cells. Pioneering studies on signaling pathways involved in pathological processes in the RPE have revealed novel molecular targets for suppressing mesenchymal differentiation. The TGF-*β*/BMP signaling pathway plays a crucial role in the mesenchymal transformation of RPE cells. The inhibition of Snail and RhoA/ROCK in the canonical and noncanonical TGF-*β* cascades reduces manifestations of mesenchymal properties in the transformed RPE cells, which offers a new approach to the prevention and treatment of PVR. Another promising therapeutic strategy consists in inhibiting mTOR, a component of the PI3K/AKT/mTOR signaling pathway. Moreover, BMP-4 signaling is regarded as a target for suppressing the effects of oxidative stress and RPE senescence in AMD. The same is true of STAT3, a component of the JAK/STAT pathway, since it plays a regulatory role in the pathogenesis of this disease. The inhibition of Notch signaling impedes EMT, retarding mesenchymal differentiation. Thus, a series of promising research approaches to control over mesenchymal differentiation of RPE cells have already taken shape. On the other hand, no less important is to find ways to stimulate and maintain neuronal differentiation of RPE with a view to restore the retina after injury or pathology. Factors operating* in vivo *restrain RPE transdifferentiation into the neural retina, but RPE cells manifest their proneural properties* in vitro.* This is of major interest for further research aimed at developing methods for retinal repair in ocular pathologies. The search for factors regulating RPE differentiation is obviously of both fundamental and practical interest. Experiments with* in vitro* cultures of human RPE allow extensive screening for changes at the cell and molecular levels that occur under pathological conditions, and profound analysis and interpretation of the results will help to find adequate approaches to correction of RPE abnormalities* in vivo*.

## Figures and Tables

**Table 1:** Human RPE cell lines (according to Mannermaa [31], modified).

Cell line	Source	References
Spontaneously transformed cell lines		
H80HrPE-6	Created by Goro Eguchi using primary RPE cells from an 80-year-old person	Tsonis et al. [32]
ARPE-19	Derived in 1986 by Amy Aotaki-Keen from the normal eyes of a 19-year-old male who died from head trauma in a motor vehicle accident	Dunn et al. [33]; ATCC CRL-2302
D407	Derived from the eye of a 12-year-old male child	Davis et al. [34]
RPE-340	Derived in 1989 from the eye of a 1-year-old female child who died from trauma	Matsunaga et al. [35]; Rambhatla et al. [36]

Immortalized cell lines		
hTERT RPE-1	Generated by transfecting the RPE-340 cell line with a plasmid expressing the human telomerase reverse transcriptase subunit (hTERT)	Rambhatla et al. [36], ATCC CRL-4000
h1RPE-7 h1RPE-116	Generated by transfecting primary RPE cells from a 50-year-old female donor with a plasmid encoding the SV40 large T antigen	Lund et al. [37] Kanuga et al. [38]

## Abbreviations

AMD: Age-related macular degeneration
BMPs: Bone morphogenetic proteins
CNV: Choroidal neovascularization
CRALBP: Cellular retinaldehyde-binding protein
ECM: Extracellular matrix
EMT: Epithelial-mesenchymal transition
ERK: Extracellular signal-regulated kinase
hTERT: Human telomerase reverse transcriptase subunit
MAPK: Mitogen-activated protein kinases
PEDF: Pigment epithelium-derived factor
PVR: Proliferative vitreoretinopathy
ROCK: Rho-associated protein kinase
RPE: Retinal pigment epithelium
RPE65: Retinal pigment epithelium-specific 65 kDa protein
TNF*α*: Tumor necrosis factor alpha
STAT: Signal transducer and activator of transcription
TER: Transepithelial electrical resistance
VEGF: Vascular endothelial growth factor.

## References

[1] Hasegawa M. (1958). Restitution of the eye after removal of the retina and lens in the newt *Triturus pyrrhogaster*. *Embryologia*.

[2] Keefe J. R. (1973). An analysis of urodelian retinal regeneration. I. Studies of the cellular source of retinal regeneration in Notophthalmus viridescens utilizing 3 H-thymidine and colchicine.. *Journal of Experimental Zoology*.

[3] Stroeva O. G., Mitashov V. I. (1983). Retinal pigment epithelium: proliferation and differentiation during development and regeneration. *International Review of Cytology*.

[4] Grigoryan E. N., Markitantova Y. V., Avdonin P. P., Radugina E. A. (2013). Study of regeneration in amphibians in age of molecular-genetic approaches and methods. *Russian Journal of Genetics*.

[5] Chiba C., Hoshino A., Nakamura K. (2006). Visual cycle protein RPE65 persists in new retinal cells during retinal regeneration of adult newt. *The Journal of Comparative Neurology*.

[6] Chiba C., Mitashov V., Chiba C. (2008). Cellular and molecular events in the adult newt retinal regeneration. *Strategies for Retinal Tissue Repair and Regeneration in Vertebrates: From Fish to Human*.

[7] Amemiya K., Haruta M., Takahashi M., Kosaka M., Eguchi G. (2004). Adult human retinal pigment epithelial cells capable of differentiating into neurons. *Biochemical and Biophysical Research Communications*.

[8] Lopez P. F., Sippy B. D., Michael Lambert H., Thach A. B., Hinton D. R. (1996). Transdifferentiated retinal pigment epithelial cells are immunoreactive for vascular endothelial growth factor in surgically excised age-related macular degeneration-related choroidal neovascular membranes. *Investigative Ophthalmology and Visual Science*.

[9] Huang X., Wei Y., Ma H., Zhang S. (2012). Vitreous-induced cytoskeletal rearrangements via the Rac1 GTPase-dependent signaling pathway in human retinal pigment epithelial cells. *Biochemical and Biophysical Research Communications*.

[10] Zhu J., Nguyen D., Ouyang H., Zhang X. H., Chen X. M., Zhang K. (2013). Inhibition of RhoA /Rho-kinase pathway suppresses the expression of extracellular matrix induced by CTGF or TGF-*β* in ARPE-19. *International Journal of Ophthalmology*.

[11] Campochiaro P. A., Hackett S. F., Conway B. P. (1991). Retinoic acid promotes density-dependent growth arrest in human retinal pigment epithelial cells. *Investigative Ophthalmology & Visual Science*.

[12] Chung E. J., Chun J. N., Jung S., Cho J. W., Lee J. H. (2011). TGF-*β*-stimulated aberrant expression of class III *β*-tubulin via the ERK signaling pathway in cultured retinal pigment epithelial cells. *Biochemical and Biophysical Research Communications*.

[13] Grierson I., Hiscott P., Hogg P., Robey H., Mazure A., Larkin G. (1994). Development, repair and regeneration of the retinal pigment epithelium. *Eye*.

[14] Grisanti S., Guidry C. (1995). Transdifferentiation of retinal pigment epithelial cells from epithelial to mesenchymal phenotype. *Investigative Ophthalmology & Visual Science*.

[15] Tamiya S., Liu L., Kaplan H. J. (2010). Epithelial-mesenchymal transition and proliferation of retinal pigment epithelial cells initiated upon loss of cell-cell contact. *Investigative Ophthalmology and Visual Science*.

[16] Hernandez E. V., Hu J. G., Frambach D. A., Gallemore R. P. (1995). Potassium conductances in cultured bovine and human retinal pigment epithelium. *Investigative Ophthalmology and Visual Science*.

[17] Chang C., Ye L., Defoe D. M., Coldwell R. B. (1997). Serum inhibits tight junction formation in cultured pigment epithelial cells. *Investigative Ophthalmology and Visual Science*.

[18] Mäenpää H., Mannerström M., Toimela T., Salminen L., Saransaari P., Tähti H. (2002). Glutamate uptake is inhibited by tamoxifen and toremifene in cultured retinal pigment epithelial cells. *Pharmacology & Toxicology*.

[19] Stanzel B. V., Espana E. M., Grueterich M. (2005). Amniotic membrane maintains the phenotype of rabbit retinal pigment epithelial cells in culture. *Experimental Eye Research*.

[20] Mannagh J., Arya D. V., Irvine A. R. (1973). Tissue culture of human retinal pigment epithelium.. *Investigative ophthalmology*.

[21] Flood M. T., Gouras P., Kjeldbye H. (1980). Growth characteristics and ultrastructure of human retinal pigment epithelium *in vitro*. *Investigative Ophthalmology and Visual Science*.

[22] Eguchi G. (1993). Lens transdifferentiation in the vertebrate retinal pigmented epithelial cell. *Progress in Retinal Research*.

[23] Feldman E. L., Del Monte M. A., Stevens M. J., Greene D. A., Jones G. E. (1996). Establishment and maintenance of in vitro cultures of human retinal pigment epithelium. *Human Cell Culture Protocols*.

[24] Stevens M. J., Larkin D. D., Feldman E. L., DelMonte M. A., Greene D. A. (2005). Establishment, maintenance, and transfection of in vitro cultures of human retinal pigment epithelium.. *Methods in molecular medicine*.

[25] Kuznetsova A. V., Milyushina L. A., Mikaelyan A. S., Zinovieva R. D., Grigoryan E. N., Aleksandrova М. A. (2010). Dedifferentiation of adult human retinal pigment epithelial cells in vitro. *Molecular Medicine*.

[26] Milyushina L. A., Kuznetsova A. V., Grigoryan E. N., Aleksandrova M. A. (2011). Phenotypic plasticity of retinal pigment epithelial cells from adult human eye in vitro. *Bulletin of Experimental Biology and Medicine*.

[27] Maminishkis A., Chen S., Jalickee S. (2006). Confluent monolayers of cultured human fetal retinal pigment epithelium exhibit morphology and physiology of native tissue. *Investigative Ophthalmology and Visual Science*.

[28] Sonoda S., Spee C., Barron E., Ryan S. J., Kannan R., Hinton D. R. (2009). A protocol for the culture and differentiation of highly polarized human retinal pigment epithelial cells.. *Nature Protocols*.

[29] Rawes V., Kipling D., Kill I. R., Faragher R. G. A. (1997). The kinetics of senescence in retinal pigmented epithelial cells: a test for the telomere hypothesis of ageing?. *Biochemistry*.

[30] Bodnar A. G., Ouellette M., Frolkis M. (1998). Extension of life-span by introduction of telomerase into normal human cells. *Science*.

[31] Mannermaa E. (2010). *In vitro model of retinal pigment epithelium for use in drug delivery studies [Dissertations in Health Sciences]*.

[32] Tsonis P. A., Jang W., Del Rio-Tsonis K., Eguchi G. (2001). A unique aged human retinal pigmented epithelial cell line useful for studying lens differentiation in vitro. *The International Journal of Developmental Biology*.

[33] Dunn K. C., Aotaki-Keen A. E., Putkey F. R., Hjelmeland L. M. (1996). ARPE-19, a human retinal pigment epithelial cell line with differentiated properties. *Experimental Eye Research*.

[34] Davis A. A., Bernstein P. S., Bok D., Turner J., Nachtigal M., Hunt R. C. (1995). A human retinal pigment epithelial cell line that retains epithelial characteristics after prolonged culture. *Investigative Ophthalmology and Visual Science*.

[35] Matsunaga H., Handa J. T., Aotaki-Keen A., Sherwood S. W., West M. D., Hjelmeland L. M. (1999). Beta-galactosidase histochemistry and telomere loss in senescent retinal pigment epithelial cells. *Investigative Ophthalmology & Visual Science*.

[36] Rambhatla L., Chiu C., Glickman R. D., Rowe-Rendleman C. (2002). In vitro differentiation capacity of telomerase immortalized human RPE cells. *Investigative Ophthalmology and Visual Science*.

[37] Lund R. D., Adamson P., Sauvé Y. (2001). Subretinal transplantation of genetically modified human cell lines attenuates loss of visual function in dystrophic rats. *Proceedings of the National Academy of Sciences of the United States of America*.

[38] Kanuga N., Winton H. L., Beauchéne L. (2002). Characterization of genetically modified human retinal pigment epithelial cells developed for in vitro and transplantation studies. *Investigative Ophthalmology and Visual Science*.

[39] Sommer F. (2006). *Hyalocytes in tissue engineering: first steps towards a cell-based vitreous substitute [Dissertation in Natural Sciences]*.

[40] Luo Y., Zhuo Y., Fukuhara M., Rizzolo L. J. (2006). Effects of culture conditions on heterogeneity and the apical junctional complex of the ARPE-19 cell line. *Investigative Ophthalmology and Visual Science*.

[41] Ma W., Song E. L., Guo J. (2007). RAGE ligand upregulation of VEGF secretion in ARPE-19 cells. *Investigative Ophthalmology and Visual Science*.

[42] Fragoso M. A., Patel A. K., Nakamura R. E. I., Yi H., Surapaneni K., Hackam A. S. (2012). The Wnt /*β*-catenin pathway cross-talks with STAT3 signaling to regulate survival of retinal pigment epithelium cells. *PLoS ONE*.

[43] Geisen P., McColm J. R., King B. M., Hartnett M. E. (2006). Characterization of barrier properties and inducible VEGF expression of several types of retinal pigment epithelium in medium-term culture. *Current Eye Research*.

[44] Chadwick B. P. (2007). Variation in Xi chromatin organization and correlation of the H3K27me3 chromatin territories to transcribed sequences by microarray analysis. *Chromosoma*.

[45] Culver-Cochran A. E., Chadwick B. P. (2012). The WSTF-ISWI chromatin remodeling complex transiently associates with the human inactive X chromosome during late S-phase prior to BRCA1 and *γ*-H2AX. *PLoS ONE*.

[46] Constable P. A., Lawrenson J. G., Dolman D. E. M., Arden G. B., Abbott N. J. (2006). P-Glycoprotein expression in human retinal pigment epithelium cell lines. *Experimental Eye Research*.

[47] Holtkamp G. M., van Rossem M., de vos A. F., Willekens B., Peek R., Kijlstra A. (1998). Polarized secretion of IL-6 and IL-8 by human retinal pigment epithelial cells. *Clinical and Experimental Immunology*.

[48] Blaauwgeers H. G. T., Holtkamp G. M., Rutten H. (1999). Polarized vascular endothelial growth factor secretion by human retinal pigment epithelium and localization of vascular endothelial growth factor receptors on the inner choriocapillaris: evidence for a trophic paracrine relation. *The American Journal of Pathology*.

[49] Hu J., Bok D. (2001). A cell culture medium that supports the differentiation of human retinal pigment epithelium into functionally polarized monolayers. *Molecular Vision*.

[50] Freshney R. I. (2005). *Culture of Animal Cells: A Manual of Basic Technique*.

[51] Martini B., Pandey R., Ogden T. E., Ryan S. J. (1992). Cultures of human retinal pigment epithelium: modulation of extracellular matrix. *Investigative Ophthalmology and Visual Science*.

[52] Tezel T. H. (1997). Reattachment to a substrate prevents apoptosis of human retinal pigment epithelium. *Graefe's Archive for Clinical and Experimental Ophthalmology*.

[53] Janssen J. J. M., Kuhlmann E. D., Van Vugt A. H. M. (2000). Retinoic acid delays transcription of human retinal pigment neuroepithelium marker genes in ARPE-19 cells. *NeuroReport*.

[54] Cai H., del Priore L. V. (2006). Gene expression profile of cultured adult compared to immortalized human retinal pigment epithelium. *Molecular Vision*.

[55] Salero E., Blenkinsop T. A., Corneo B. (2012). Adult human RPE can be activated into a multipotent stem cell that produces mesenchymal derivatives. *Cell Stem Cell*.

[56] Burke J. M., Skumatz C. M. B., Irving P. E., McKay B. S. (1996). Phenotypic heterogeneity of retinal pigment epithelial cells in vitro and in situ. *Experimental Eye Research*.

[57] Ablonczy Z., Dahrouj M., Tang P. H. (2011). Human retinal pigment epithelium cells as functional models for the RPE in vivo. *Investigative Ophthalmology and Visual Science*.

[58] Hu J., Bok D. (2010). Culture of highly differentiated human retinal pigment epithelium for analysis of the polarized uptake, processing, and secretion of retinoids. *Methods in Molecular Biology*.

[59] Kannan R., Zhang N., Sreekumar P. G. (2006). Stimulation of apical and basolateral vascular endothelial growth factor-A and vascular endothelial growth factor-C secretion by oxidative stress in polarized retinal pigment epithelial cells. *Molecular Vision*.

[60] Ohno-Matsui K., Morita I., Tombran-Tink J. (2001). Novel mechanism for age-related macular degeneration: an equilibrium shift between the angiogenesis factors VEGF and PEDF. *Journal of Cellular Physiology*.

[61] Blenkinsop T. A., Salero E., Stern J. H., Temple S. (2013). The culture and maintenance of functional retinal pigment epithelial monolayers from adult human eye. *Methods in Molecular Biology*.

[62] Hay E. D. (2005). The mesenchymal cell, its role in the embryo, and the remarkable signaling mechanisms that create it. *Developmental Dynamics*.

[63] Ahlstrom J. D., Erickson C. A. (2009). The neural crest epithelial-mesenchymal transition in 4D: a “tail” of multiple non-obligatory cellular mechanisms. *Development*.

[64] Akhurst R. J., Derynck R., Miyazono K. (2007). TGF-*β* Signaling in Epithelial-Mesenchymal Transition and Invasion and Metastasis. *The TGF-beta Family*.

[65] Nabi I. R., Mathews A. P., Cohen-Gould L., Gundersen D., Rodriguez-Boulan E. (1993). Immortalization of polarized rat retinal pigment epithelium. *Journal of Cell Science*.

[66] Marrs J. A., Andersson-Fisone C., Jeong M. C. (1995). Plasticity in epithelial cell phenotype: modulation by expression of different cadherin cell adhesion molecules. *Journal of Cell Biology*.

[67] Alge C. S., Suppmann S., Priglinger S. G. (2003). Comparative proteome analysis of native differentiated and cultured dedifferentiated human RPE cells. *Investigative Ophthalmology and Visual Science*.

[68] McKay B. S., Irving P. E., Skumatz C. M. B., Burke J. M. (1997). Cell-cell adhesion molecules and the development of an epithelial phenotype in cultured human retinal pigment epithelial cells. *Experimental Eye Research*.

[69] Burke J. M., Cao F., Irving P. E., Skumatz C. M. B. (1999). Expression of E-cadherin by human retinal pigment epithelium: delayed expression in vitro. *Investigative Ophthalmology and Visual Science*.

[70] van Aken E. H., de Wever O., van Hoorde L., Bruyneel E., de Laey J., Mareel M. M. (2003). Invasion of retinal pigment epithelial cells: N-cadherin, hepatocyte growth factor, and focal adhesion kinase. *Investigative Ophthalmology and Visual Science*.

[71] Maeda M., Johnson K. R., Wheelock M. J. (2005). Cadherin switching: essential for behavioral but not morphological changes during an epithelium-to-mesenchyme transition. *Journal of Cell Science*.

[72] Imamichi Y., Menke A. (2007). Signaling pathways involved in collagen-induced disruption of the E-cadherin complex during epithelial-mesenchymal transition. *Cells Tissues Organs*.

[73] McKechnie N. M., Boulton M., Robey H. L., Savage F. J., Grierson I. (1988). The cytoskeletal elements of human retinal pigment epithelium: *in vitro* and *in vivo*. *Journal of Cell Science*.

[74] Ruiz A., Brett P., Bok D. (1996). TIMP-3 is expressed in the human retinal pigment epithelium. *Biochemical and Biophysical Research Communications*.

[75] Kigasawa K., Ishirawa H., Obazawa H., Minamoto T., Nagai Y., Tanaka Y. (1998). Collagen production by cultured human retinal pigment epithelial cells. *Tokai Journal of Experimental and Clinical Medicine*.

[76] Newsome D. A., Pfeffer B. A., Hewitt A. T., Robey P. G., Hassell J. R. (1988). Detection of extracellular matrix molecules synthesized in vitro by monkey and human retinal pigment epithelium: Influence of donor age and multiple passages. *Experimental Eye Research*.

[77] Philp N. J., Nachmias V. T. (1987). Polarized distribution of integrin and fibronectin in retinal pigment epithelium. *Investigative Ophthalmology and Visual Science*.

[78] Campochiaro P. A., Sugg R., Grotendorst G., Hjelmeland L. M. (1989). Retinal pigment epithelial cells produce PDGF-like proteins and secrete them into their media. *Experimental Eye Research*.

[79] Edwards R. B. (1982). Glycosaminoglycan synthesis by cultured human retinal pigmented epithelium from normal postmortem donors and a postmortem donor with retinitis pigmentosa. *Investigative Ophthalmology and Visual Science*.

[80] Stramm L. E. (1987). Synthesis and secretion of glycosaminoglycans in cultured retinal pigment epithelium. *Investigative Ophthalmology and Visual Science*.

[81] Kim Y. S., Jung D. H., Kim N. H., Lee Y. M., Kim J. S. (2007). Effect of magnolol on TGF-*β*1 and fibronectin expression in human retinal pigment epithelial cells under diabetic conditions. *European Journal of Pharmacology*.

[82] Lee J., Ko M., Joo C. (2008). Rho plays a key role in TGF-*β*1-induced cytoskeletal rearrangement in human retinal pigment epithelium. *Journal of Cellular Physiology*.

[83] Moll R., Franke W. W., Schiller D. L., Geiger B., Krepler R. (1982). The catalog of human cytokeratins: patterns of expression in normal epithelia, tumors and cultured cells. *Cell*.

[84] Sheridan C., Hiscott P., Grierson I., Kirchhof B., Wong D. (2005). Retinal pigment epithelium differentiation and dedifferentiation. *Essentials in Ophthalmology: Vitreo-Retinal Surgery*.

[85] Engelhardt M., Bogdahn U., Aigner L. (2005). Adult retinal pigment epithelium cells express neural progenitor properties and the neuronal precursor protein doublecortin. *Brain Research*.

[86] Vinores S. A., Derevjanik N. L., Mahlow J. (1995). Class III *β*-tubulin in human retinal pigment epithelial cells in culture and in epiretinal membranes. *Experimental Eye Research*.

[87] Milyushina L. A., Verdiev B. I., Kuznetsova A. V., Aleksandrova M. A. (2012). Expression of multipotent and retinal markers in pigment epithelium of adult human in vitro. *Bulletin of Experimental Biology and Medicine*.

[88] Kuznetsova A. V., Grigoryan E. N., Aleksandrova M. A. (2011). Human adult retinal pigment epithelial cells as potential cell source for retina recovery. *Cell and Tissue Biology*.

[89] Cacci E., Villa A., Parmar M. (2007). Generation of human cortical neurons from a new immortal fetal neural stem cell line. *Experimental Cell Research*.

[90] Andersson E. R., Sandberg R., Lendahl U. (2011). Notch signaling: Simplicity in design, versatility in function. *Development*.

[91] Vaisman N. Y. (2003). Cell signaling pathways in animal ontogeny. *VOGiS Herald*.

[92] Moustakas A., Kowanetz M., Thuault S., Dijke P., Heldin C.-H. (2006). TGF-*β*/SMAD signaling in epithelial to mesenchymal transition. *Smad Signal Transduction: Smads in Proliferation, Differentiation and Disease. Proteins and Cell Regulation*.

[93] Mitsuhiro M. R. K., Eguchi S., Yamashita H. (2003). Regulation mechanisms of retinal pigment epithelial cell migration by the TGF-*β* superfamily. *Acta Ophthalmologica Scandinavica*.

[94] Xu J., Lamouille S., Derynck R. (2009). TGF-*Β*-induced epithelial to mesenchymal transition. *Cell Research*.

[95] Li H., Wang H., Wang F., Gu Q., Xu X. (2011). Snail involves in the transforming growth factor *β*1-mediated epithelial-mesenchymal transition of retinal pigment epithelial cells. *PLoS ONE*.

[96] Dudley A. T., Lyons K. M., Robertson E. J. (1995). A requirement for bone morphogenetic protein-7 during development of the mammalian kidney and eye. *Genes and Development*.

[97] Luo G., Hofmann C., Bronckers A. L. J. J., Sohocki M., Bradley A., Karsenty G. (1995). BMP-7 is an inducer of nephrogenesis, and is also required for eye development and skeletal patterning. *Genes and Development*.

[98] Furuta Y., Hogan B. L. M. (1998). BMP4 is essential for lens induction in the mouse embryo. *Genes and Development*.

[99] Müller F., Rohrer H., Vogel-Höpker A. (2007). Bone morphogenetic proteins specify the retinal pigment epithelium in the chick embryo. *Development*.

[100] Wordinger R. J., Clark A. F. (2007). Bone morphogenetic proteins and their receptors in the eye. *Experimental Biology and Medicine*.

[101] Mathura J. R., Jafari N., Chang J. T. (2000). Bone morphogenetic proteins-2 and-4: negative growth regulators in adult retinal pigmented epithelium. *Investigative Ophthalmology and Visual Science*.

[102] Zhu D., Deng X., Xu J., Hinton D. R. (2009). What determines the switch between atrophic and neovascular forms of age related macular degeneration?—the role of BMP4 induced senescence.. *Aging*.

[103] Zhu D., Wu J., Spee C., Ryan S. J., Hinton D. R. (2009). BMP4 mediates oxidative stress-induced retinal pigment epithelial cell senescence and is overexpressed in age-related macular degeneration. *Journal of Biological Chemistry*.

[104] Xu J., Zhu D., He S., Spee C., Ryan S. J., Hinton D. R. (2011). Transcriptional regulation of bone morphogenetic protein 4 by tumor necrosis factor and its relationship with age-related macular degeneration. *The FASEB Journal*.

[105] Lee K., Nelson C. M. (2012). New insights into the regulation of epithelial-mesenchymal transition and tissue fibrosis. *International Review of Cell and Molecular Biology*.

[106] Amano M., Fukata Y., Kaibuchi K. (2000). Regulation and functions of Rho-associated kinase. *Experimental Cell Research*.

[107] Loirand G., Guérin P., Pacaud P. (2006). Rho kinases in cardiovascular physiology and pathophysiology. *Circulation Research*.

[108] Lutty G. A., Merges C., Threlkeld A. B., Crone S., McLeod D. S. (1993). Heterogeneity in localization of isoforms of TGF-*β* in human retina, vitreous, and choroid. *Investigative Ophthalmology and Visual Science*.

[109] Connor T. B., Roberts A. B., Sporn M. B. (1989). Correlation of fibrosis and transforming growth factor-*β* type 2 levels in the eye. *Journal of Clinical Investigation*.

[110] Potekhina E. S., Nadezhdina E. S. (2002). Mitogen-activated protein kinase cascades and the involvement of Ste20-like protein kinases in them. *Uspekhi Biologicheskoi Khimii*.

[111] Chen X., Xiao W., Wang W., Luo L., Ye S., Liu Y. (2014). The complex interplay between ERK1/2, TGF*β*/Smad, and Jagged/Notch signaling pathways in the regulation of epithelial- mesenchymal transition in retinal pigment epithelium cells. *PloS ONE*.

[112] Galy A., Néron B., Planque N., Saule S., Eychène A. (2002). Activated MAPK/ERK kinase (MEK-1) induces transdifferentiation of pigmented epithelium into neural retina. *Developmental Biology*.

[113] Saika S., Yamanaka O., Ikeda K. (2005). Inhibition of p38MAP kinase suppresses fibrotic reaction of retinal pigment epithelial cells. *Laboratory Investigation*.

[114] Zhao C., Yasumura D., Li X. (2011). mTOR-mediated dedifferentiation of the retinal pigment epithelium initiates photoreceptor degeneration in mice. *Journal of Clinical Investigation*.

[115] Nagineni C. N., Kutty V., Detrick B., Hooks J. J. (2005). Expression of PDGF and their receptors in human retinal pigment epithelial cells and fibroblasts: Regulation by TGF-*β*. *Journal of Cellular Physiology*.

[116] Lei H., Rheaume M., Kazlauskas A. (2010). Recent developments in our understanding of how platelet-derived growth factor (PDGF) and its receptors contribute to proliferative vitreoretinopathy. *Experimental Eye Research*.

[117] Hollborn M., Tenckhoff S., Jahn K. (2005). Changes in retinal gene expression in proliferative vitreoretinopathy: glial cell expression of HB-EGF. *Molecular Vision*.

[118] Chen Y. J., Tsai R. K., Wu W. C., He M. S., Kao Y., Wu W. S. (2012). Enhanced PKC*δ* and ERK signaling mediate cell migration of retinal pigment epithelial cells synergistically induced by HGF and EGF. *PLoS ONE*.

[119] Takahashi E., Nagano O., Ishimoto T. (2010). Tumor necrosis factor-*α* regulates transforming growth factor-*β*-dependent epithelial-mesenchymal transition by promoting hyaluronan-CD44-moesin interaction. *Journal of Biological Chemistry*.

[120] Liu Y., Cao G., Xue J. (2012). Tumor necrosis factor-alpha (TNF-*α*)-mediated in vitro human retinal pigment epithelial (RPE) cell migration mainly requires Akt/mTOR complex 1 (mTORC1), but not mTOR complex 2 (mTORC2) signaling. *European Journal of Cell Biology*.

[121] Wang C., Cao G., Jiang Q., Yao J. (2012). TNF-*α* promotes human retinal pigment epithelial (RPE) cell migration by inducing matrix metallopeptidase 9 (MMP-9) expression through activation of Akt/mTORC1 signaling. *Biochemical and Biophysical Research Communications*.

[122] Defoe D. M., Grindstaff R. D. (2004). Epidermal growth factor stimulation of RPE cell survival: Contribution of phosphatidylinositol 3-kinase and mitogen-activated protein kinase pathways. *Experimental Eye Research*.

[123] Clevers H. (2006). Wnt/*β*-Catenin Signaling in Development and Disease. *Cell*.

[124] Gregory-Evans C., Wallace V. A., Gregory-Evans K. (2013). Gene networks: dissecting pathways in retinal development and disease. *Progress in Retinal and Eye Research*.

[125] Nelson W. J., Nusse R. (2004). Convergence of Wnt, *β*-catenin, and cadherin pathways. *Science*.

[126] Gavert N., Ben-Ze'ev A. (2007). *β*-Catenin signaling in biological control and cancer. *Journal of Cellular Biochemistry*.

[127] Thompson M. D., Monga S. P. S. (2007). WNT/*β*-catenin signaling in liver health and disease. *Hepatology*.

[128] Burke J. M. (2008). Epithelial phenotype and the RPE: is the answer blowing in the Wnt?. *Progress in Retinal and Eye Research*.

[129] Rak D. J., Hardy K. M., Jaffe G. J., McKay B. S. (2006). Ca++-switch induction of RPE differentiation. *Experimental Eye Research*.

[130] Kwon H., Lee H., Ji Y., Rubin J. S., Tomarev S. I. (2009). Myocilin is a modulator of Wnt signaling. *Molecular and Cellular Biology*.

[131] Zhang S. S., Liu M., Kano A., Zhang C., Fu X., Barnstable C. J. (2005). STAT3 activation in response to growth factors or cytokines participates in retina precursor proliferation. *Experimental Eye Research*.

[132] Fasler-Kan E., Wunderlich K., Hildebrand P., Flammer J., Meyer P. (2005). Activated STAT3 in choroidal neovascular membranes of patients with age-related macular degeneration. *Ophthalmologica*.

[133] Schouwey K., Larue L., Radtke F., Delmas V., Beermann F. (2010). Transgenic expression of Notch in melanocytes demonstrates RBP-J*κ*-dependent signaling. *Pigment Cell and Melanoma Research*.

[134] Bao Z., Cepko C. L. (1997). The expression and function of notch pathway genes in the developing rat eye. *Journal of Neuroscience*.

[135] Lee H. Y., Wroblewski E., Philips G. T. (2005). Multiple requirements for Hes1 during early eye formation. *Developmental Biology*.

[136] Tomita K., Ishibashi M., Nakahara K. (1996). Mammalian hairy and Enhancer of split homolog 1 regulates differentiation of retinal neurons and is essential for eye morphogenesis. *Neuron*.

[137] Hatakeyama J., Bessho Y., Katoh K. (2004). Hes genes regulate size, shape and histogenesis of the nervous system by control of the timing of neural stem cell differentiation. *Development*.

[138] Schouwey K., Aydin I. T., Radtke F., Beermann F. (2011). RBP-J*κ*-dependent Notch signaling enhances retinal pigment epithelial cell proliferation in transgenic mice. *Oncogene*.

[139] Saad S., Stanners S. R., Yong R., Tang O., Pollock C. A. (2010). Notch mediated epithelial to mesenchymal transformation is associated with increased expression of the Snail transcription factor. *International Journal of Biochemistry and Cell Biology*.

[140] Chen X. (2014). Blockade of jagged/notch pathway abrogates transforming growth factor *β*2-induced epithelial-mesenchymal transition in human retinal pigment epithelium cells. *Current Molecular Medicine*.

[141] Schouwey K., Beermann F. (2008). The Notch pathway: hair graying and pigment cell homeostasis. *Histology and Histopathology*.

[142] Saika S., Yamanaka O., Okada Y. (2009). TGF*β* in fibroproliferative diseases in the eye. *Frontiers in Bioscience—Scholar*.

[143] Yan X., Liu Z., Chen Y. (2009). Regulation of TGF-*β* signaling by Smad7. *Acta Biochimica et Biophysica Sinica*.

[144] Ishida W., Mori Y., Lakos G. (2006). Intracellular TGF-*β* receptor blockade abrogates smad-dependent fibroblast activation *in vitro* and *in vivo*. *Journal of Investigative Dermatology*.

[145] Li J., Campanale N. V., Liang R. J., Deane J. A., Bertram J. F., Ricardo S. D. (2006). Inhibition of p38 mitogen-activated protein kinase and transforming growth factor-*β*1/smad signaling pathways modulates the development of fibrosis in adriamycin-induced nephropathy. *The American Journal of Pathology*.

[146] Pannu J., Nakerakanti S., Smith E., Ten Dijke P., Trojanowska M. (2007). Transforming growth factor-*β* receptor type I-dependent fibrogenic gene program is mediated via activation of Smad1 and ERK1/2 pathways. *Journal of Biological Chemistry*.

[147] Hou Q., Tang J., Wang Z. (2013). Inhibitory effect of microRNA-34a on retinal pigment epithelial cell proliferation and migration. *Investigative Ophthalmology & Visual Science*.

[148] Favata M. F., Horiuchi K. Y., Manos E. J. (1998). Identification of a novel inhibitor of mitogen-activated protein kinase kinase. *Journal of Biological Chemistry*.

[149] Nakamura K., Nishimura J., Hirano K., Ibayashi S., Fujishima M., Kanaide H. (2001). Hydroxyfasudil, an active metabolite of fasudil hydrochloride, relaxes the rabbit basilar artery by disinhibition of myosin light chain phosphatase. *Journal of Cerebral Blood Flow and Metabolism*.

[150] Gao Y., Dickerson J. B., Guo F., Zheng J., Zheng Y. (2004). Rational design and characterization of a Rac GTPase-specific small molecule inhibitor. *Proceedings of the National Academy of Sciences of the United States of America*.

[151] Itoh Y., Kimoto K., Imaizumi M., Nakatsuka K. (2007). Inhibition of RhoA/Rho-kinase pathway suppresses the expression of type I collagen induced by TGF-beta2 in human retinal pigment epithelial cells. *Experimental Eye Research*.

[152] Becerra E. M., Morescalchi F., Gandolfo F. (2012). Clinical evidence of intravitreal triamcinolone acetonide in the management of age-related macular degeneration. *Current Drug Targets*.

[153] Xiao W., Chen X., Liu X., Luo L., Ye S., Liu Y. (2014). Trichostatin A, a histone deacetylase inhibitor, suppresses proliferation and epithelial-mesenchymal transition in retinal pigment epithelium cells. *Journal of Cellular and Molecular Medicine*.

[154] Fernández-Robredo P., Sancho A., Johnen S. (2014). Current treatment limitations in age-related macular degeneration and future approaches based on cell therapy and tissue engineering. *Journal of Ophthalmology*.

